# The Comparative Toxicity, Biochemical and Physiological Impacts of Chlorantraniliprole and Indoxacarb on *Mamestra brassicae* (Lepidoptera: Noctuidae)

**DOI:** 10.3390/toxics11030212

**Published:** 2023-02-24

**Authors:** Moataz A. M. Moustafa, Eman A. Fouad, Emad Ibrahim, Anna Laura Erdei, Zsolt Kárpáti, Adrien Fónagy

**Affiliations:** 1 Department of Economic Entomology and Pesticides, Faculty of Agriculture, Cairo University, Giza 12613, Egypt; 2 Department of Bioassay, Central Agricultural Pesticides Laboratory, Agricultural Research Center, Giza 12618, Egypt; 3 Plant Virus and Vector Interactions, Crop Research Institute, 16106 Prague, Czech Republic; 4 Plant Protection Institute, Centre for Agricultural Research, Eötvös Lóránd Research Network (ELKH), 1022 Budapest, Hungary; 5 Department of Plant Protection Biology, Swedish University of Agricultural Sciences, 23053 Uppsala, Sweden; 6 Animal Ecology and Tropical Biology, University of Würzburg, 97070 Würzburg, Germany

**Keywords:** toxicity, sublethal effects, chlorantraniliprole, indoxacarb, *Mamestra brassicae*

## Abstract

Background: The cabbage moth, *Mamestra brassicae,* is a polyphagous pest that attacks several crops. Here, the sublethal and lethal effects of chlorantraniliprole and indoxacarb were investigated on the developmental stages, detoxification enzymes, reproductive activity, calling behavior, peripheral physiology, and pheromone titer of *M. brasssicae.* Methods: To assess pesticide effects, the second instar larvae were maintained for 24 h on a semi-artificial diet containing insecticides at their LC_10_, LC_30_, and LC_50_ concentrations. Results: *M. brassicae* was more susceptible to chlorantraniliprole (LC_50_ = 0.35 mg/L) than indoxacarb (LC_50_ = 1.71 mg/L). A significantly increased developmental time was observed with both insecticides at all tested concentrations but decreases in pupation rate, pupal weight, and emergence were limited to the LC_50_ concentration. Reductions in both the total number of eggs laid per female and the egg viability were observed with both insecticides at their LC_30_ and LC_50_ concentrations. Both female calling activity and the sex pheromone (Z11-hexadecenyl acetate and hexadecenyl acetate) titer were significantly reduced by chlorantraniliprole in LC_50_ concentration. Antennal responses of female antennae to benzaldehyde and 3-octanone were significantly weaker than controls after exposure to the indoxocarb LC_50_ concentration. Significant reductions in the enzymatic activity of glutathione *S*-transferases, mixed-function oxidases, and carboxylesterases were observed in response to both insecticides.

## 1. Introduction

The cabbage moth *Mamestra brassicae* Linnaeus (Lepidoptera: Noctuidae) is a highly polyphagous insect pest that currently threatens more than 300 plant species, most of which belong to the Brassicaceae species [1,2]. Generally, this pest annually accounts for around 20–25% of damage in vegetables and 25–40% of yield decreases [3]. In cases of heavy infestation, *M. brassicae* can cause significant losses (up to 50%) in cabbage crops, especially under warm and humid conditions that allow the larvae to continuously feed on the aboveground parts of the plant in the night and morning hours [4,5,6]. Historically, chemical insecticides were used intensively to control economically important insect pest outbreaks. However, this overreliance on pesticide intervention has resulted in the development of resistance [7]. Consequently, there is a growing need for new types of insecticides that might delay or prevent the development of resistance. Two classes of insecticides developed by DuPont de Nemours to exhibit some of these characteristics are diamides and oxadiazine. To date, both chemical classes have been used against a broad range of insect pests from different orders, including Coleoptera, Diptera, Hemiptera, Isoptera, and Lepidoptera [7,8,9,10,11,12,13,14]. Moreover, these insecticides, which appear to be safe for humans and other non-target organisms (e.g., mammals, fish, beneficial insects, and mites), have highly selective modes of action and thus immense potential for use in integrated pest management programs [15,16,17,18,19,20].

Chlorantraniliprole is a novel anthranilic diamide insecticide that binds to and modulates insect ryanodine receptor function [18]. The ryanodine receptor regulates the activity of intracellular channels that release Ca^2+^ into muscles. The perturbation of ryanodine receptor function in insects can result in the excessive release of Ca^2+^, resulting in lethargy, muscle paralysis, and death [18]. Given this mode of action, chlorantraniliprole has the potential to become one of the most promising agents in resistance management [21]. Indoxacarb is another non-traditional insecticide that belongs to the oxadiazine insecticide group and is bioactivated to a decarbomethoxylated metabolite by insect esterases and/or amidases [22,23]. Indoxacarb acts by blocking the movement of Na^+^ ions into nerve cells causing nervous system shutdown, paralysis, and death of the targeted pests [14,24].

Knowledge of the lethal and sublethal effects of insecticides is critical for successful biological control and chemical applications [25]. Sublethal effects are defined as physiological, biological, and behavioral changes in individual insects or populations [26]. More recently, the toxicity and sublethal impacts of chlorantraniliprole and indoxacarb were reported in several insect pests [27,28,29,30,31,32,33,34,35]. Sublethal effects might also reflect protective physiological responses such as the complement of cytochrome P450-dependent monooxygenases, carboxylesterases (CarE), and glutathione *S*-transferases (GST) that impact insecticide metabolism [36]. The induction of these detoxification enzymes following insecticide exposure can limit insecticide efficacy by increasing their metabolism and/or secretion [37].

The toxicity and physiological impacts of chlorantraniliprole and indoxacarb on *M. brassicae* have not yet been documented. Therefore, in the present work, the lethal and sub-lethal effects of both insecticides on *M. brassicae* development, larval detoxification enzyme activity, reproductive activity, olfactory physiology, calling behavior, and pheromone titer were investigated.

## 2. Materials and Methods

### 2.1. Mamestra brassicae Culture

The *M. brassicae* stock colony was maintained in a rearing room at 25 ± 1 °C and 60% relative humidity. To harmonize with working hours, reversed photoperiod conditions of 8D:16L (dark began at 8:00 to 16:00) were used. A semi-artificial diet was offered for larval feeding [38,39]. Pupae collected from the soil were sexed and kept separately in 25 × 15 × 8 cm plastic containers furnished with a layer of tissue paper. Adults were housed separately in cylindrical jars (12 × 10 cm) covered with a fine mesh. Sterilized 10% honey solution was applied on a piece of cotton and the jars were furnished with folded brown paper for shelter [39]. Newly emerged adults were defined as day 0 (D0). Post-emergence days were similarly defined through D6. For scotophase monitoring (i.e., calling behavior), adults were kept under the same conditions as above in a room equipped with a dim red light [30,39].

### 2.2. Insecticides

The tested insecticides were chlorantraniliprole (Coragen^®^ 20% suspension concentrate, DuPont, Saint Laurent Du Pont, France), and indoxacarb (Avaunt^®^ 15%, suspension concentrate, DuPont, France).

### 2.3. Bioassays

Insecticidal activities of chlorantraniliprole and indoxacarb were tested on early second instar *M. brassicae* larvae. A diet-overlay method was performed [40] in small plastic cylindrical cups (6 cm in diameter and 6 cm in height) in which soft food was pressed on the bottom of the cup to a height of approximately 1 cm. A 250 µL aliquot of both insecticides at various concentrations [4, 2, 1, 0.5, 0.05, and 0.005 mg/L (ppm)] was spread on the surface and allowed to permeate the diet for an hour before the larvae were placed into the cups. Each concentration was tested on 150 larvae across three replicates. The larvae were allowed to feed for 24 h on the treated diet. Then, the alive larvae were transferred onto an insecticide-free diet. Mortality was recorded after four days post-treatment and the sublethal and lethal concentrations of each insecticide were determined [30,41]. Each bioassay was replicated twice.

### 2.4. Sublethal and Lethal Effects

#### 2.4.1. Effects of Chlorantraniliprole and Indoxacarb on Development

Sublethal and lethal concentrations corresponding to the respective LC_10_, LC_30_, and LC_50_ concentrations for chlorantraniliprole and indoxacarb were administered to second instar larvae and were used to determine the effects on the duration of larval and pupal development, mortality, and adult emergence [30,41]. The number of days to complete larval development was recorded daily until the last instar, which was transferred to clean cups containing autoclaved soil for pupation. After 6 days, each pupa was sexed, weighed, and then returned to the original cup but with moist cotton swabs for humidity. The pupal duration and percentage of adults that successfully emerged from the pupal stage were recorded.

#### 2.4.2. Fecundity and Fertility

After second instar larvae were exposed to the LC_10_, LC_30_, and LC_50_ concentrations, surviving adults were separated into groups of 5 females and 6–7 males per replicate for egg deposition [31,39]. Each group was placed underneath brown paper in glass jars (12 × 10 cm) containing folded brown paper and cotton bulbs soaked in a 10% honey solution. The jar was covered with a fine cloth, as described above. Egg batches were counted daily for 6 days with the percentage of eggs that hatched determined. Each concentration was assessed across three replicates.

### 2.5. Effects on Larval Detoxification Enzymes

#### 2.5.1. Sample Preparation

At 4 days postsecond instar treatment with the insecticide at LC_10_, LC_30_, and LC_50_ concentrations, surviving larvae were pooled and homogenized (0.05 g larvae/replicate) in cold 0.1 M phosphate buffer at pH 7.4 for mixed-function oxidases (MFO), pH 7.0 for carboxylesterase (CarE), or pH 6.5 for glutathione *S*-transferase (GST) activity. The homogenate was centrifuged for 15 min at 7000× *g*. The supernatants were transferred directly to clean and sterilized Eppendorf tubes (1.5 mL) for biochemical analysis.

#### 2.5.2. MFO Activity

MFO activity was tested according to Hansen and Hodgson [42] and Moustafa et al. [30]. The larval homogenate was incubated for 2 min at 27 °C with *p*-nitro anisole (2 mM), and then NADPH (9.6 mM) was added to start the reaction. MFO activity was measured at 405 nm using a microplate reader (Clindiag-MR-96, ISO09001:2008, Steenberg, Belgium). The MFO activity was calculated based on a *p*-nitrophenol standard curve.

#### 2.5.3. CarE Activity

CarE activity (including α- and ß-esterase) was determined according to Van Asperen [43] and Moustafa et al. [30]. The larval homogenate was incubated with α- or ß-naphthyl acetate (30 mM) at 25 °C for 15 min. The reaction was terminated by adding 50 µL of stop solution [Fast Blue b (2%): sodium dodecyl sulphate (5%)]. The hydrolysis of α-naphthyl acetate was measured at 600 nm, while ß-naphthyl acetate was measured at 550 nm on a Jenway-7205UV/Vis Spectrophotometer, Staffordshire, UK. The CarE activity was calculated based on α- and ß-naphthyl acetate standard curves.

#### 2.5.4. GST Activity

GST activity was determined as described by Habig et al. [44] and Moustafa et al. [30]. The reaction solution contained the larval homogenate, CDNB (30 mM), and GSH (50 mM). The reaction was measured at 340 nm at 25 °C for 3 min on a Jenway-7205 UV/Vis Spectrophotometer, Staffordshire, UK.

### 2.6. Effect on Calling Behavior

The calling behavior of virgin female moths was recorded according to Moustafa et al. [30] using D1-D5 adults derived from second instar larvae treated with the insecticide at LC_10_, LC_30_, and LC_50_ concentrations. Observations were performed at one-hour intervals during scotophase, from 8:00 to 16:00, in an experimental room equipped with a dim red light. Data for 6 females (cumulative across 5 days) for each concentration were recorded. Based on the pheromone gland (PG) protrudence, female responses were determined as either calling (protruded PG) or non-calling (PG that was not visible).

### 2.7. Analysis of Pheromone Blends

#### 2.7.1. Preparation of Pheromone Gland Extract

For pheromone gland blend analysis (characterization and quantification), PGs were excised from D2 females between the 6th and 8th h of scotophase, similarly as performed by Moustafa et al. [30]. The PGs were extracted separately in glass extraction vials containing 150 μL *n*-hexane for approximately 10 min and then the extract was transferred to a conical glass insert (200 μL) that was then placed into a 1.5 mL vial suitable for an automatic sampler coupled to the Gas Chromatography–Mass Spectroscopy (GC-MS) unit. An internal standard, *E*11-tetradecenyl acetate (500 ng/5 μL; Pherobank BV, The Netherlands), was added and the extract was concentrated to approximately 20 μL using a thermos block at 60 °C. The vials were stored at −30 °C until needed.

#### 2.7.2. Gas Chromatography–Mass Spectrometry Analysis

A 1 μL aliquot of each sample was injected via an automatic splitless mode into a GC-MS unit (Hewlett Packard GC 6890, HP MSD 5973) equipped with a RESTEC (Rxi-5SI) column (0.25 mm internal diameter × 30 m and 0.25 μm film thickness) using helium (6.00) as a carrier gas at a flow rate of 1 mL/min. The running and heating conditions used were as described in Moustafa et al. [39] and Hull et al. [45]. The Selective Ion Method (SIM) was used for the rapid but sensitive fractionation of the main (*Z*11-hexadecenyl acetate) and minor (hexadecenyl acetate) pheromone components. Quantification was performed using MSD Chemstation ver. D.01.02.16. An authentic standard (*E*11-tetradecenyl acetate) was injected in scan mode to build the standard curve using *Z*11-hexadecenyl acetate and hexadecenyl acetate (Pherobank BV, The Netherlands) for six representative concentrations.

### 2.8. Electrophysiological Recordings

To assess the antennal responses of female and male cabbage moths to different host plant odors and pheromone components, peripheral electroantennographic recordings (EAGs) were conducted, as described by Molnár et al. [46]. Briefly, we excised the antenna of the moth and cut the last distal segment. The proximal side of the antenna was inserted into a glass capillary (ID 1.17 mm, Syntech, Kirchzarten, Germany) filled with Ringer’s solution [47]. The capillary with the antenna was attached to the silver reference electrode. The distal side of the antenna was inserted into another glass capillary filled with Ringer’s solution and connected to the recording electrode. During the recordings, the antennal signal was amplified 10× and converted to a digital signal using a signal acquisition interface (IDAC-2, Syntech). The converted signal was recorded on GC-EAD software (GC-EAD 2014, version 1.2.5, Syntech). During the antennal recordings, the antenna was placed into a charcoal-filtered, humidified air stream (1 L/min). The synthetic odorants were selected according to Jacquin et al. [48], Ulland et al. [49], and Wei et al. [50]. Odorants were dissolved in *n*-hexane and 10 μL of a 1 μg/μL dilution was deposited on filter paper (1 × 1 cm) and placed into a Pasteur pipette as the stimulus cartridge. The stimulation time was 0.5 s and the stimulation airflow, generated with a Stimulus Controller (CS-55, Syntech), was 2 L/min. For the control stimulus *n*-hexane was used. The control stimulus was applied to the antenna at the start and end of each stimulus regime. The first and last control stimuli were averaged, and this value was subtracted from the values obtained for all other stimuli.

### 2.9. Statistical Analysis

The corrected *M. brassicae* larval mortality percentages were statistically analyzed according to Finney [51] using Probit analysis (LDP-line software; http://www.ehabsoft.com/ldpline/DownloadForm.htm; accessed on 21 January 2020) to estimate the sublethal and lethal values (LC_10_, LC_30_, and LC_50_) of chlorantraniliprole and indoxacarb at 4 days post-treatment. To investigate the effects of the LC_10_, LC_30_, and LC_50_ insecticide concentrations, statistical analyses were performed using Statistica v 13.0 (Statsoft Inc., Tulsa, OK, USA). Assumptions of normality and homogeneity of variance were assessed using Shapiro–Wilk and Levene’s tests, respectively. The results were analyzed usingthe one-way ANOVA and Tukey post-hoc tests. When data were not normally distributed and/or variances were unequal, a Kruskal-Wallis test was performed followed by multiple comparisons of mean ranks. Statistical significance was set at *p* < 0.05. The percentages of pupation, sex ratio, emergence, and hatch were transformed via Arc sine [52] prior to the statistical analyses. The same software was used to assess the calling behavior.

## 3. Results

### 3.1. Toxicity of Chlorantraniliprole and Indoxacarb on M. brassicae

Bioassay results for both chlorantraniliprole and indoxacarb against second instar *M. brassicae* larvae revealed that chlorantraniliprole was more toxic than indoxacarb (Table 1). The chlorantraniliprole LC_10_, LC_30_, and LC_50_ values were 0.001, 0.03, and 0.35 mg/L, respectively, whereas the LC values of indoxacarb were 0.08, 0.50, and 1.71 mg/L, respectively (Table 1).

### 3.2. Effects of Chlorantraniliprole and Indoxacarb on M. brassicae Biological Parameters

#### 3.2.1. *M. brassicae* Life Table

Effects of the sublethal and lethal LC_10_, LC_30_, and LC_50_ concentrations for both tested insecticides on the life table parameters of *M. brassicae* are presented in Table 2. Chlorantraniliprole and indoxacarb significantly prolonged the duration of both the larval and pupal stages under all tested concentrations (Table 2). In contrast, female pupal weight was significantly decreased in the LC_50_ experimental groups for both insecticides (Table 2). Only indoxacarb (LC_50_ concentration) had an effect on male pupal weight (Table 2). No significant differences were found in the pupation percentage (Table 2) or the sex ratio of the experimental groups (Table 3). However, the percentage of adult emergence was significantly reduced in the indoxacarb (LC_50_ concentration) group compared to the control group (F = 3.40; *p* = 0.027) (Table 3).

#### 3.2.2. Fecundity and Fertility

Both the sublethal and lethal concentrations of chlorantraniliprole and indoxacarb reduced female fecundity compared to the control group: the reduction in eggs laid per female was 1.04-, 1.14-, and 1.37-fold lower for chlorantraniliprole and 1.23-, 1.34-, and 1.44-fold for indoxacarb at the LC_10_, LC_30_, and LC_50_ concentrations, respectively (Table 3). No significant differences were found in the percentage of eggs that hatched (hatchability percentage) in either of the experimental groups (Table 3).

### 3.3. Effects on Larval Detoxification Enzymes

The activities of multiple detoxification enzymes (MFO, CarE, and GST) were significantly reduced in all of the experimental treatment groups (Table 4). MFO activities were much lower in the indoxacarb LC_30_ and LC_50_ groups, whereas CarE and GST activities were lowest in the indoxacarb LC_50_ group.

### 3.4. Effect on Calling Behaviour

**Figure 1:**
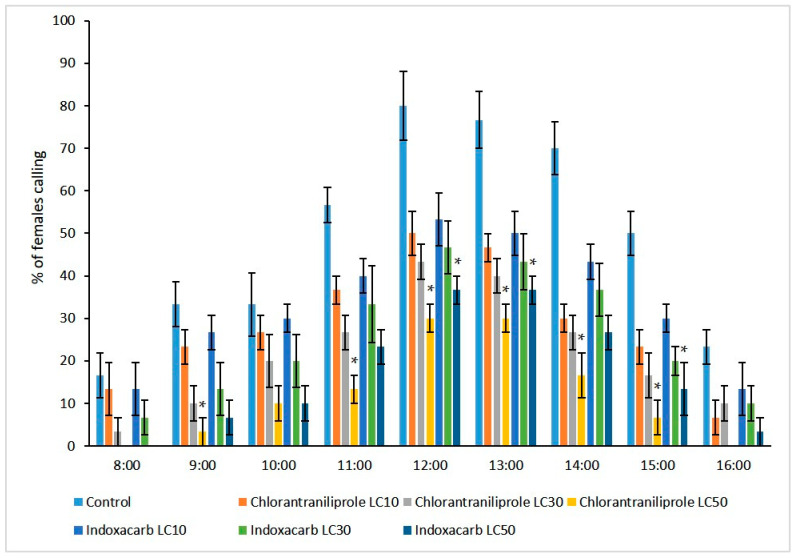
The effect of treatments on the calling behavior of adult *M. brassicae* females.

Calling activity was highest between the 5th (12:00) and 7th (14:00) hours of scotophase (Figure 1). For chlorntraniliprole, female calling in the LC_10_, LC_30_, and LC_50_ groups was 50.0 ± 5.27% (F = 10.97, *p* ≤ 0.0001), 43.33 ± 4.08% (F = 9.43, *p* ≤ 0.0001), and 30.0 ± 3.33% (F = 11.37, *p* ≤ 0.0001), respectively. Similar results were seen in the indoxacarbLC_10_, LC_30_, and LC_50_ groups with 53.33 ± 6.23% (F = 8.76, *p* ≤ 0.0001), 46.67 ± 6.23% (F = 6.02, *p* ≤ 0.0001), and 36.66 ± 3.33% (F = 12.56, *p* ≤ 0.0001), respectively (Figure 1). In contrast, a significantly higher percentage of females (80.0 ± 8.16%) exhibited calling behavior in the control group.

The percentage of females exhibiting calling behavior ± SE (n = 6) was recorded between D1-D5 *M. brassicae* females during scotophase (8 h from 8:00 to 16:00). Asterisks indicate significant differences within that specific time point as compared to the control group at *p* < 0.05 (Kruskal-Wallis test).

### 3.5. Effects on Pheromone Production

Changes in two pheromone components (Z11-16Ac and 16Ac) of D2 virgin *M. brassicae* females were determined using GC-MS after treating second instar larvae with chlorantraniliprole or indoxacarb at their LC_10_, LC_30_, and LC_50_ concentrations. In Table 5, the amounts (ng/PG) of the two components are listed according to their retention time. Indoxacarb had no effect and chlorantraniliprole effects varied depending on the concentration with the lowest sublethal concentration accentuating production (i.e., higher than the control) and the LC_50_ concentration nearly reducing by 50% (Table 5).

### 3.6. Electroantennographic Recordings

**Figure 2:**
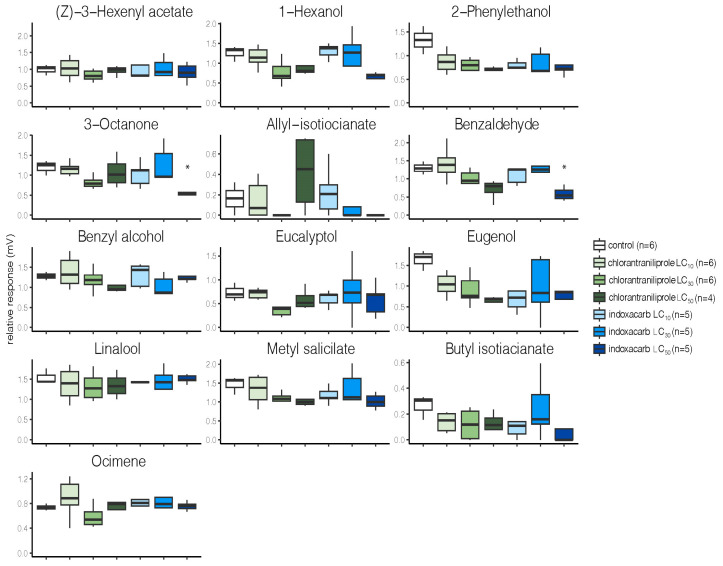
Electroantennographic recordings of female *M. brassicae.* In box plots, center lines indicate the median values, the box limits indicate the 25th and 75th percentiles, and the whiskers extend 1.5 times the interquartile range from the 25th and 75th percentiles. The averaged control stimulus (*n*-hexane) measured before and after the stimulus regime was subtracted from the absolute EAG amplitudes for each compound. Asterisk indicates a significant difference to the control at *p*  <  0.05 (Kruskal-Wallis test). The sources of the chemicals are as follows: ***Z***-3-hexenyl acetate (Jena, Germany); 1-hexanol (Sigma, Darmstadt, Germany); 2-phenylethanol (Sigma, Germany); 3-octanone (SASRI); allyl isotiocinate (Sigma, Germany); benzadehyde (BB); benzyl alcohol (Sigma, Germany); eucalyptol (Sigma Aldricht, Germany; eugenol (SASRI); linalool (Sigma, Germany); metyl salycilate (Jena, Germany); butyl isotiacianate (Alnarp, Sweden); and ocimene, rcemic (Jena, Germany).

**Figure 3:**
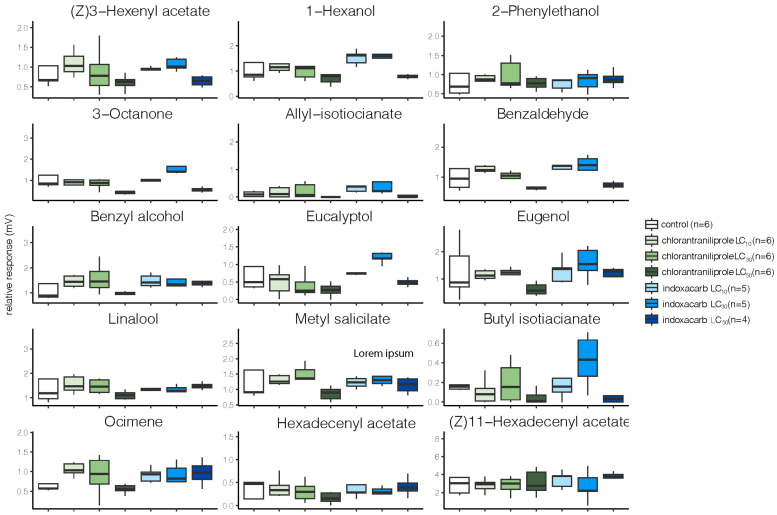
Electroantennographic recordings of male *M. brassicae.* In box plots, center lines indicate the median values, the box limits indicate the 25th and 75th percentiles, and the whiskers extend 1.5 times the interquartile range from the 25th and 75th percentiles. The averaged control stimulus (*n*-hexane) was measured before and after the stimulus regime and was subtracted from the absolute EAG amplitudes for each compound. No significant differences were found in any of the experimental groups. See the sources of the compounds in Figure 2 legends and text.

The EAG recordings showed that exposure of second instar *M. brassicae* larvae to varying concentrations of the two insecticides had minimal effects on the ability of adults of either sex to detect plant volatiles or pheromone components (Figure 2 and Figure 3). Among the 13 odorants tested, a significant decrease in the relative antennal response was only observed with benzaldehyde and 3-octanone in females from the indoxacarb LC_50_ group (Figure 2). Chlorantraniliprole had no effects on female odorant detection. For males, no significant effects were seen in any of the experimental groups (Figure 3).

## 4. Discussion

Exposure of larvae to a sublethal concentration of insecticide does not necessarily lead to the death of the target pest. Rather, it may affect biochemical reactions and physiological processes [30,32,41,53] that eventually modify biological parameters such as life cycle, reproduction, and/or duration of development, all of which singly or collectively can have negative effects on insect population dynamics [25,37]. Therefore, evaluating the sublethal effects of insecticides on different insect stages including larvae and adults [54,55], and applying that knowledge to pest management practices, can help maintain insecticide efficacy by delaying the development of insecticide resistance. Furthermore, knowledge of insect demographic parameters can provide insights into the optimal approach for control. Consequently, the main objective of this study was to investigate the effects of sublethal concentrations of the insecticides chlorantraniliprole and indoxcarb on *M. brassicae* demographic parameters, namely behavior, pheromone production and activity of known larval detoxification enzymes.

In this study, second instar *M. brassicae* larvae were more susceptible to chlorantraniliprole than to indoxacarb, which is consistent with a previous report [56] that found that a laboratory strain of *Spodoptera exigua* Hübner (Lepidoptera: Noctuidae) was more susceptible to chlorantraniliprole (LC_50_ = 0.014 mg/L) than 18 different field strains in China. Furthermore, a laboratory strain of *Helicoverpa armigera* Hübner (Lepidoptera: Noctuidae) was more susceptible to chlorantraniliprole (LC_50_ = 0.0147 μg/mL) than indoxacarb (LC_50_ = 0.147 μg/mL) [57]. *H. armigera* third instar larvae had a high LC_50_ (5.93 μg/mL) for indoxacarb [58], while second instar *Spodoptera littoralis* Boisd. (Lepidoptera: Noctuidae) larvae were reported to be more tolerant to indoxacarb than chlorantraniliprole [30].

All treatments with sublethal concentrations of both insecticides increased the number of days necessary to complete both larval and pupal development in *M. brassicae*. Likewise, the larval duration of *S. littoralis* was extended following exposure to the LC_25_ concentration of both insecticides [59]. Furthermore, sublethal concentrations of these insecticides significantly increased the length of larval and pupal duration in *S. littoralis* [30]. The pupal period of *Plutella xylostella* Linnaeus (Lepidoptera: Plutellidae) was significantly extended by the S- and R-indoxacarb groups as compared to controls [60]. Sublethal treatment with indoxcarb also significantly increased the duration of the larval and pupal stages in *H. armigera* and reduced pupal weight [37].

Sublethal doses of insecticides can influence detoxification enzymes as a protective response, as our results indicate that both insecticides significantly reduced ß-esterase and GST activities but had no significant effect on MFO. Similarly, Vojoudi et al. [37] reported that indoxcarb (LC_30_) reduced carboxylesterase and GST activities at 72 h post-treatment, while CarE and GST activities were increased 24 h post-treatment in third instar *H. armigera* larvae. In contrast, the sublethal concentrations of chlorantraniliprole and indoxacarb increased MFO, GST, and CarE activities in second instar *S. littoralis* larvae [30]. A study of the toxicity metabolism induced in *S. frugiperda* after sublethal (LC_10_ concentration) exposure to chlorantraniliplore found differential expression of 1266 genes with 578 up-regulated and 688 down-regulated. Exposure to the LC_30_ concentration resulted in differential expression of 3637 genes with 1545 up-regulated and 2092 down-regulated [61].

The highest calling activity in our experimental *M. braccicae* groups was found 2–4 h prior to the end of scotophase. In contrast, calling behavior was observed between the 2nd and 4th h of scotophase after treatment with sublethal concentrations of bioinsecticides [39]. This shift in the calling time peak has also been reported in other lepidoteran insect pests after bioinsecticide treatment [61,62]. This alteration could be due to the type of insecticide used and/or the mode of action.

Both sex pheromone biosynthesis and production in moths involve a tightly coordinated physiological process that is under hormonal control and which is typically regulated by a neuropeptide termed pheromone biosynthesis activating neuropeptide [63,64]. Peak pheromone production in noctuids [65] and *M. braccicae* [39] generally occurs towards the end of scotophase. The results of pheromone blend (*Z*11-hexadecenyl acetate and hexadecenyl acetate) analysis indicate that our sublethal insecticidal treatment of second instar *M. braccicae* larvae had both negative and positive effects on some of the pheromone components (Table 5). A non-significant decrease was recorded in the chlorantraniliprole LC_50_ group, whereas an increase was found in the LC_10_ group. However, a significant decrease was detected in an earlier study when second instar *M. brassicae* larvae were treated with different sublethal concentrations of spinosad and evamectin benzoate [39]. In *Ostrinia furnacalis* Guenee (Lepidoptera: Carmbidae), it has been found that females as first and third instar larvae treated with deltamethrin had significantly higher amounts of pheromone biosynthesis [66], which resulted in a broader ratio of the blend components.

Fecundity and insect fertility rates are potentially affected by insecticide exposure. These parameters can be utilized in pest management to predict future population sizes [67] because reductions in the number of offspring can drop pest populations below economic threshold levels. Our results indicated that fecundity and fertility were negatively affected by sublethal concentrations of chlorantraniliprole and indoxacarb relative to controls. This is consistent with reports that indoxacarb reduces the fecundity and fertility of *H. armigera* [37]. Furthermore, indoxacarb reduced *P. xylostella* fertility [68], whereas sublethal concentrations of chlorantraniliprole and indoxcarb reduced fecundity and fertility in *P. xylostella, Tuta absoluta* Meyrick (Lepidoptera: Gelechiidae), and *Spodoptera litura* Fabricius (Lepidoptera: Noctuidae) [54,60,69].

*M. brassicae* olfaction is essential for finding mate partners and potential oviposition sites. Our electrophysiological antennae recordings indicated that female antennae in the indoxacarb LC_50_ group were less responsive to benzaldehyde and 3-octanone. Interestingly, males’ antennal responses to the odorants and pheromone components were not affected significantly. These differences between females and males could be due to the Larval indoxacarb treatment likewise having no effect on pheromone detection in *P. xylostella* adult males [70]. However, Wu et al. [71] found a significant reduction in odor detection in the oriental fruit fly (*Bactrocera dorsalis* Hendel; Diptera: Tephritidae) after β-cypermethrin exposure. A two-choice olfactometer study similarly showed that imidacloprid reduced the ability of female *Nasonia vitripennis* Walker (Hymenoptera: Pteromalidae) parasitoid wasps to detect male sex pheromones and find hosts [72]. In our study, weakened *M. brassicae* female antennae responses were limited to benzaldehyde and 3-octanone; detection of the other odorants remained normal. This impairment could be due to reduced olfactory receptor expression. In addition to decreased antennal responses, *B. dorsalis* also exhibited altered chemoreceptor expression after β-cypermethrin treatment [71]. However, the insecticide treatment in *B. dorsalis* did not suppress the expression of all of the chemoreceptors, which could explain the differential odor detection we observed in the *M. brassicae* females. Alternatively, males and females might respond differently to insecticide treatment such that reduced olfactory receptor expression only occurs in females with no effect on male odor detection. Reduced olfactory detection of host plant volatiles via insecticide treatment could impact foraging and host plant finding, i.e., females are unable to locate their host plants, resulting in decreased oviposition. As an indirect pest control process, insecticide treatment reduces the number of laid eggs on the host plant, resulting in fewer larvae and less damage to the plant.

## 5. Conclusions

In summary, chemical insecticides have frequently been used to control lepidopteran insect pests. However, their efficacy may decline as a result of insecticide-resistant populations. Consequently, the continued success of management practices is dependent on the development and introduction of new insecticides such as chlorntraniliprole and indoxacarb. This study indicates that both chlorntraniliprole and indoxacarb have insecticidal activities and biochemical and physiological impacts against *M. brassicae*. However, further investigations are underway to evaluate their side effects on the non-target organisms.

## Figures and Tables

**Table 1:** Toxicity of chlorantraniliprole and indoxacarb on second instar *M. brassicae* larvae. The tested insecticides were applied at 4, 2, 1, 0.5, 0.05, and 0.005 mg/L (ppm) to the diet surface. Each concentration assay was replicated three times using 150 s instar larvae per replicate. The larvae were allowed to feed for 24 h on a treated diet and were then transferred onto an untreated diet. Sublethal and lethal concentrations were determined 4 days post-treatment.

Insecticides	LC_10_ (mg/L) 95% Confidence Limits	LC_30_ (mg/L) 95% Confidence Limits	LC_50_ (mg/L) 95% Confidence Limits	Slope ± SE	χ^2^	*p*
Chlorantraniliprole	0.001 (0.0001–0.005)	0.03 (0.01–0.08)	0.35 (0.13–0.61)	0.52 ± 0.08	5.04	0.40
Indoxacarb	0.08 (0.01–0.19)	0.50 (0.22–0.84)	1.71 (1.03–3.51)	0.98 ± 0.20	2.28	0.51

**Table 2:** Effects of chlorantraniliprole and indoxacarb on the development of larval and pupal stages in *M. brassicae* (mean ± SE). Insects were treated as second instar larvae with different concentrations of insecticides. Durations are indicated as days, pupation %, and weight in mg (mean ± SE).

		Larval Duration (Days)	Pupal Duration (Days)	Pupation %	Pupal Weight (mg)	
Treatments					Female	Male
Control		19.3 ± 0.2 a	22.0 ± 0.3 a	95.0 ± 2.6	364 ± 17 a	352 ± 20 a
Chlorantaniliprole	LC_10_	21.1 ± 0.3 b	24.4 ± 0.3 b	86.14 ± 7.9	325 ± 23 ab	325 ± 16 ab
	LC_30_	22.0 ± 0.3 bc	25.6 ± 0.4 b	83.33 ± 9.6	289 ± 14 abc	299 ± 17 ab
	LC_50_	22.3 ± 0.4 bc	26.1 ± 0.5 b	81.8 ± 11.6	264 ± 12 bc	271 ± 11 ab
Indoxacarb	LC_10_	22.0 ± 0.3 bc	24.2 ± 0.6 b	92.59 ± 7.4	279 ± 11 abc	269 ± 12 ab
	LC_30_	22.6 ± 0.2 c	25.2 ± 0.5 b	90.55 ± 5.8	293 ± 14 abc	275 ± 13 ab
	LC_50_	22.8 ± 0.3 c	26.2 ± 0.6 b	80.0 ± 5.1	231 ± 17 c	225 ± 17 b

Means within a column followed by different letters are significantly different (*p* < 0.05). Where there is no letter indicated in that respective column, no significant difference was found.

**Table 3:** Effects of chlorantraniliprole and indoxacarb on adult emergence, sex ratio, fecundity, and hatchability in *M. brassicae* (mean ± SE). Insects were treated as second instar larvae with different concentrations of insecticides.

Treatment		Emergence (%)	Males (%)	Fecundity	Hatchability (%)
Control		94.5 ± 2.8 a	54.6 ± 2.9	326.9 ± 21.5	93.7 ± 3.2
Chlorantraniliprole	LC_10_	95.0 ± 2.6 a	51.5 ± 10.12	310.8 ± 41.7	89.4 ± 2.4
	LC_30_	88.9 ± 5.9 ab	52.8 ± 4.9	285.1 ± 70.1	88.2 ± 2.8
	LC_50_	79.3 ± 2.0 ab	45.7 ± 5.7	236.2 ± 8.1	85.3 ± 3.3
Indoxacarb	LC_10_	88.1 ± 6.5 ab	51.6 ± 12.1	264.7 ± 56.9	87.0 ± 1.8
	LC_30_	79.5 ± 5.7 ab	51.9 ± 9.3	242.1 ± 18.4	86.3 ± 2.8
	LC_50_	67.2 ± 8.7 b	46.0 ± 6.5	225.6 ± 23.7	77.9 ± 6.5

Means within a column followed by different letters are significantly different (*p* < 0.05). Where there is no letter indicated in that respective column, no significant difference was found.

**Table 4:** Activities of detoxification enzymes (mean ± SE), such as mixed-function oxidases (MFO), carboxylesterase (CarE), and glutathione S-trasnsferase (GST), in second instar larvae of *M. brassicae* four days after chlorantraniliprole or indoxacarb treatments.

Treatment		MFO (nmole/min/mg Protein)	CarE (nmole/min/mg Protein)		GST (μmole/min/mg Protein)
			α-Esterases	β-Esterases	
Control		16.3 ± 0.92 ab	176 ± 10	565 ± 25 a	25.5 ± 1.46 a
Chlorantraniliprole	LC_10_	26.1 ± 6.63 a	122 ± 20	538 ± 37 ab	18.1 ± 0.91 ab
	LC_30_	13.8 ± 0.80 ab	123 ± 15	498 ± 65 abc	16.6 ± 1.65 ab
	LC_50_	14.7 ± 1.31 ab	117 ± 9	341 ± 13 bcd	14.4 ± 0.75 ab
Indoxacarb	LC_10_	14.5 ± 1.35 ab	150 ± 16	431 ± 71 abc	17.1 ± 0.92 ab
	LC_30_	12.0 ± 0.95 ab	145 ± 5	317 ± 11 cd	15.6 ± 1.13 ab
	LC_50_	11.2 ± 0.06 b	115 ± 12	204 ± 14 d	12.2 ± 0.15 b

Means within a column followed by different letters are significantly different (*p* < 0.05). Where there is no letter indicated in that respective column, no significant difference was found.

**Table 5:** Effects of chlorantraniliprole or indoxacarb treatments on sex pheromone titers (ng/female ± SE, n = 7) of 2-day-old *M. brassicae* females (at the 5th and 6th hour of scotophase). Insects were treated as 2nd instar larvae with different concentrations of insecticides.

Treatments		*Z*11-Hexadecenyl Acetate	Hexadecenyl Acetate
Control		259.6 ± 37.5 ab	11.7 ± 1.8
Chlorantraniliprole	LC_10_	326.4 ± 22.4 a	16.7 ± 1.0
	LC_30_	250.2 ± 50.6 ab	12.8 ± 2.2
	LC_50_	166.3 ± 29.7 b	8.7 ± 1.5
Indoxacarb	LC_10_	247.8 ± 31.7 ab	13.1 ± 2.2
	LC_30_	261.9 ± 73.6 ab	15.4 ± 6.2
	LC_50_	280.5 ± 20.9 ab	15.6 ± 1.6

Means within a column followed by different letters are significantly different (*p* < 0.05). Where there is no letter indicated in that respective column, no significant difference was found.

## Data Availability

All data of the study have been presented in the manuscript.
